# Prevalence and Risk Factors of Musculoskeletal Disorders Among Clinical Laboratory Technicians

**DOI:** 10.3390/healthcare13121406

**Published:** 2025-06-12

**Authors:** Rawan Aldhabi, Ahmed Alzahrani, Mashael Alsobhi, Majed Albadi, Saad Alfawaz, Umar Alabasi, Muataz Almaddah, Afnan Gmmash, Ziyad Neamatallah, Riziq Allah Gaowgzeh

**Affiliations:** 1 Department of Physical Therapy, Faculty of Medical Rehabilitation Sciences, King Abdulaziz University, Jeddah 21589, Saudi Arabia; mgalsobhi@kau.edu.sa (M.A.); malbadi@kau.edu.sa (M.A.); saadlfawaz@kau.edu.sa (S.A.); ualabasi@kau.edu.sa (U.A.); malmaddah@kau.edu.sa (M.A.); asgmmsh@kau.edu.sa (A.G.); zneamatallah@kau.edu.sa (Z.N.); rizikjoresearch@gmail.com (R.A.G.); 2 Department of Physical Therapy, King Faisal Medical Complex, Taif 26514, Saudi Arabia; ahmed.pt.ksa@gmail.com

**Keywords:** musculoskeletal pain, ergonomics risk factors, laboratory technician

## Abstract

**Introduction:** Musculoskeletal disorders (MSDs) are a significant occupational health concern worldwide, particularly among healthcare professionals such as laboratory technicians. MSDs lead to chronic pain, decreased productivity, and reduced quality of life. This study aimed to investigate the prevalence of MSDs and associated ergonomics risk factors among Saudi clinical laboratory technicians. **Methodology**: This cross-sectional study was conducted on 167 clinical laboratory technicians in Taif city, Saudi Arabia. Data were collected through an online self-administered questionnaire, distributed via Google Forms. The questionnaire collected demographics information, assessed the prevalence of musculoskeletal pain using the Nordic Musculoskeletal Questionnaire (NMQ), and evaluated ergonomics risk factors using the Dutch Musculoskeletal Questionnaire (DMQ). **Results**: In total, 77.3% of the sample exhibited musculoskeletal issues in the last 12 months, with lower back (52.1%), neck (48.5%), and shoulders (40.7%) being the most frequent muscular complaints among laboratory technicians. Experience and nationality showed significant associations with MSDs (*p* ≤ 0.05). Lower back and neck complaints were commonly recorded with multiple laboratory tasks, including sustained sitting and standing and repetitive movement, whereas lower back and shoulder pain were notably prevalent with pipetting work. **Conclusions**: Work-related musculoskeletal disorders were highly apparent in laboratory practice. Periodic ergonomic training is mandated among laboratory personnel to limit occupational disability.

## 1. Introduction

Musculoskeletal disorders (MSDs) represent a significant health concern, affecting the muscles, tendons, ligaments, joints, cartilage, nerves, and intervertebral disks [1]. These disorders are particularly prevalent in work-related settings, capturing the attention of healthcare and insurance organizations [2]. MSDs often manifest in pain, paresthesia, and weakness, impacting a wide range of occupations, including healthcare professionals [3]. The MSDs in the healthcare sector alone impact millions of workers and cost public health systems and companies billions of dollars with over two million missed workdays [4]. Work-related risk factors contributing to MSDs include insufficient breaks, extended work shifts, repetitive movements, poor postures, static muscle strain, excessive arm and hand exertion, sudden muscular movements, high cognitive demands, lower job satisfaction, and anxiety, which further exacerbate MSD-related pain and discomfort [5,6,7].

Furthermore, clinical laboratory technicians are particularly vulnerable to MSDs due to the physical demands of their work. Tasks such as pipetting, handling microscopes, using bioanalyzers, and other repetitive activities involve prolonged bending of the neck, twisting, wrist strain, thumb pressure, pinch grips, standing for extended periods, and overreaching, all of which increase the risk of developing work-related MSDs [7]. Despite these risks, the health and safety of laboratory technicians in their workplace are often overlooked, with limited implementation of preventive measures and insufficient emphasis on occupational health policies.

Previous studies have reported an overall MSD prevalence ranging from 40 to 60% among medical laboratory professionals globally, with the neck being the most commonly affected region (18–78%) [8]. Despite the high prevalence and significant risk factors of MSDs, limited research has been conducted to address the burden of MSDs in this specific population in Saudi Arabia. Therefore, this study aimed to investigate the prevalence of MSDs and their associated ergonomics risk factors among clinical laboratory technicians in Taif city, Saudi Arabia. This study aims to address the gap in the current literature and provide guidance on improving workplace ergonomics to reduce the prevalence of MSDs in this population.

## 2. Methodology

### 2.1. Study Design and Participants

This cross-sectional study was conducted among clinical laboratory technicians working in both government and private hospitals in Taif city, Saudi Arabia, from March 2021 to July 2021. Participants were recruited through word-of-mouth, and electronic survey forms were distributed within laboratory departments. The inclusion criteria were male and female clinical laboratory technicians who have fixed working hours with a minimum of 8 h per day. Participants with a history of orthopedic disease or musculoskeletal trauma in the past 6 months to their participation were excluded. Prior to participation, all eligible participants received a detailed explanation of the study’s purpose and procedures and signed an informed consent. Ethical approval was obtained by the Ethical Review Board of the College of Medical Rehabilitation Sciences, King Abdulaziz University (reference # FMRS-EC2021-19).

#### Sample Size

G*Power (version 3.1) for a chi-square test of independence was used to determine the minimum sample size required to detect a medium effect size of (w = 0.3) with an alpha level of 0.05, and a power of 0.80. The required minimum sample size was calculated to be 133 participants. In this study, the sample size of 167 exceeds the recommended threshold, indicating that the study was sufficiently powered.

### 2.2. Data Collection Methods and Procedure

Data were collected through an online self-administered questionnaire using Google Forms. The questionnaire consisted of three sections: The first section was used to collect data regarding sociodemographic information, including age, gender, height, weight, educational level, and number of years of work experience. The second section included the validated Nordic Musculoskeletal Questionnaire (NMQ) to evaluate the prevalence of MSDs among the participants. The NMQ assesses if participants had experienced any pain in any of the nine anatomical regions of their bodies (neck, shoulder, upper back, elbows, lower back, wrist/hands, hips/thighs, knees, and feet/ankles), specifically in the year before the survey and in the seven days prior to the study, and if they were unable to perform their jobs because of pain in the same nine body sites. A body diagram was included in the survey to aid participants in identifying the affected anatomical regions [9].

The third section of the survey utilized the Dutch Musculoskeletal Questionnaire (DMQ) [10] to assess various ergonomics factors, including both dynamic and static load, standing and sitting postures, repetitive movements, and overall working conditions. It also focused on specific ergonomic considerations, such as the design of computer workstations and the use of certain laboratory equipment, such as microtomes, pipettes, and microscopes.

### 2.3. Statistical Analysis

Data analysis was performed using the Statistical Package for the Social Sciences (SPSS) version 26. Descriptive statistics, including frequencies and percentages, were used to summarize categorical data, while continuous data were presented as means and standard deviations. A chi-square test was used to assess the association between variables. Statistical significance was defined as a *p*-value less than 0.05.

## 3. Results

### 3.1. Participants Characteristics

A total of 205 medical laboratory technicians filled out the questionnaire, with 167 included in the final analysis after excluding those with a medical history of orthopedic diseases or musculoskeletal trauma. Most of the participants were female (n = 87, 52.1%) and Saudi nationals (n = 143, 85.6%). The age distribution indicated that most technicians were between 20 and 40 years old (n = 151, 90.4%). Regarding professional experience, 32.3% (n = 54) had over 10 years of experience, while 30.5% (n = 51) had less than a year. The body mass index (BMI) categorization showed that 47.3% (n = 79) of participants were within the healthy range (18.5 < BMI < 24.9), 32% (n = 54) were overweight (25 < BMI < 29.9), 16% (n = 27) were classified as obese (BMI ≥ 30), and 4.2% (n = 7) were underweight (BMI < 18.5). Table 1 displays the detailed sociodemographic attributes of the participants.

### 3.2. Prevalence of MSDs

Regarding the overall prevalence of MSDs among laboratory technicians, 58.1% of the participants reported experiencing ache, pain, discomfort, or numbness in at least one site of the nine body sites within the last 7 days, while 71.3% indicated having muscular discomfort over the last 12 months.

In the past 12 months, the most prevalent MSDs complaint was reported at the lower back (52.1%), followed by the neck (48.5%), shoulders (40.7%), upper back (33.5%), knees (32.3%), and ankles/feet (31.1%). The participants reported less prevalent MSD troubles in the wrists/hands (28.1%), hips/thighs (16.8%), and elbows (13.2%). In contrast, for the last 7 days, participants indicated MSDs issues predominately in the neck (37.5%), lower back (33.5%), shoulders (25.7%), and upper back (21%), with fewer complaints occurring regarding the ankles/feet (19.8%), knees (18.6%), wrists/hands (12.6%), hips/thighs (9%), and elbows (7.2%) (Table 2).

### 3.3. Factors Associated with Work-Related Musculoskeletal Disorders

Table 3 presents the results of the chi-square test indicating the significant factors of sociodemographic and occupational characteristics, and the presence of MSDs in various body parts in the last 12 months among laboratory technicians. The results showed that MSD prevalence had a significant association with nationality (*p* = 0.04) and work experience (*p* = 0.02). The prevalence of MSDs in Saudis was higher compared to non-Saudi participants. In addition, the results revealed that among 119 participants who experienced MSDs in the past year, 88.2% of them had 5 to 10 years of experience. On the other hand, MSDs prevalence had a non-significant association with gender (*p* = 0.43), age (*p* = 0.34), and BMI (*p* = 0.21).

Table 4 shows that standing for long periods, sitting for long periods, working in the same posture for long periods, and performing tasks which require awkward position, arm/hand exertion, and repetitive movements were significantly associated with MSDs among laboratory technicians. Out of 119 participants who reported MSDs, 87 participants stand for long periods, while participants who sit for long periods experienced less MSDs issues (n = 65). The results indicated working in the same posture for long periods induced MSDs among laboratories (n = 75).

**Figure 1:**
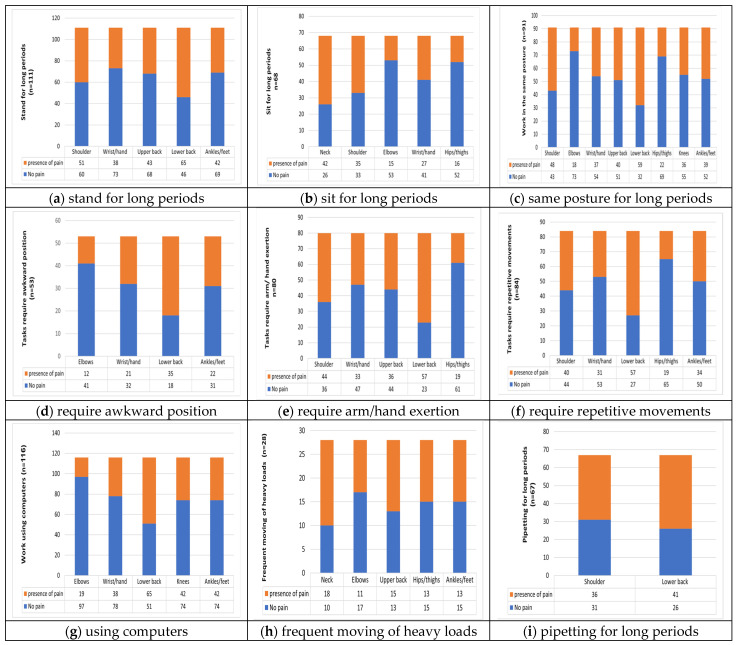
(**a**–**i**) The association between the nature of laboratory work and the prevalence of MSDs in different body regions.

Figure 1 illustrates the association between the nature of laboratory work and the prevalence of MSDs in different body regions. A statistically significant relationship was found only between certain job roles and reported musculoskeletal pain.

## 4. Discussion

MSDs are evident and identified as a significant burden on individuals and, eventually, the national economy among healthcare professionals [11]. This study aimed to explore the prevalence of MSDs and identify the potential risk factors among clinical laboratory technicians in Taif City, Saudi Arabia. The overall 12-month prevalence of muscular disorders among laboratory technicians at any anatomical site was found to be 71.3%, which is similar to previously published studies that found MSD manifestations were high among laboratory workers [7,12,13,14,15,16,17]. In the medical laboratory field, the high prevalence of muscle discomfort could be attributed to the frequent repetitive motions associated with microscopy, pipetting, and microtomy performed by laboratory personnel. The prolonged sitting or standing on those repetitive movements could result in muscle, joint, tendon, or nerve pain [7], potentially leading to occupational disability [18]. This raises the necessity of ergonomic awareness among laboratory staff.

The present study identified lower back, neck, and shoulder pain as the primary complaints of discomfort reported by laboratory technicians with a prevalence rate of 52.1%, 48.5%, and 40.7%, respectively. These results align with prior studies investigating work-related musculoskeletal pain in the healthcare sector. The predominant complaint of lower back pain in this survey is consistent with data from laboratory staff in several countries [12,15,19,20,21] that highlighted back pain as a common muscular issue among this population. Furthermore, neck and shoulder pain was highly reported by participants, which is similar to data found in the literature [7,22]. However, the findings of the lowest percentage of muscular pain in the wrists/hands (28.1%), hips/thighs (16.8%), and elbows (13.2%) were in line with previous studies [15,23], attributable to the nature of laboratory work that slightly strains these body regions.

Given the increased susceptibility of laboratory personnel to MSDs, it is essential to discover the leading factors. In this study, MSDs were found to be significantly associated with nationality (*p* = 0.04) and work experience (*p* = 0.02). In contrast, previous studies reported nationality to have no significant relationship with muscular discomfort among laboratory workers [14,22]. Numerous studies have highlighted the existence of associations between cultural differences and MSD prevalence among workers from various nationalities in countries such as Korea [24], Thailand [25], and the United States [26]. Considering that the study’s design limits causal inferences, it is imperative to investigate the possible impact of cultural and workplace factors to improve the occupational health policies. This resulted in future research recommendations for a more profound understanding of these factors among the technician population, employing different study designs, such as longitudinal or qualitative approaches. The disagreement with the findings might be related to the small sample size and the high proportion of the national participants in the survey.

In regard to work experience, it was documented as a key factor that is significantly associated with MSDs in laboratory settings, corroborated by several research that revealed a high prevalence of muscular illness among experienced employees [15,19]. This may be attributed to the sustained and ergonomically demanding postures associated with specific laboratory tasks such as microscopy or pipetting. Although these tasks are estimated to occupy approximately 300 h annually, they involve repetitive movements and static loading that can contribute disproportionately to musculoskeletal discomfort and injury risk [8].

On the other hand, MSD incidence exhibited no significant association with gender. This finding was inconsistent with previous studies that reported a substantial association with gender, indicating more muscle discomfort among female laboratory professionals [14,21,22]. This discrepancy might be attributed to the nature of the occupational tasks, which are most likely to be the same among both genders in laboratory settings, leading to minimizing the gender-based variations in MSD prevalence. Further research is required for an in-depth explanation the relationship between MSDs and gender. Generally, this study showed a higher prevalence of MSD symptoms among females compared to males. Gender differences might be attributed to the biological and physical variations between both genders. Moreover, age and BMI were not indicators of MSDs, which is in line with previous findings of no association with muscular disorders [19]. Nevertheless, other studies indicated a significant relationship between age, BMI, and the presence of MSDs [15,21,27]. This might be attributed to diminished muscular mass and strength, which were reported to be correlated with aging [28]. In terms of BMI, Agrawal et al. [8], discussed the existing association of BMI with muscle disorders among medical laboratory professionals. Further research is suggested to investigate this phenomenon with a wider age range and bigger sample size to justify the discrepancies in the results in the literature.

Exploring the relationship between MSDs and job nature is imperative because of the possibility of laboratory practices to incorporate ergonomic risks. This study revealed that maintaining sustained standing or sitting postures in awkward or improper positions leads to musculoskeletal discomfort among workers. Prior research concluded analogous results of various muscular pains, indicating that prolonged sitting and standing are associated with muscular disorder, with neck and lower back pain being the most prevalent among the laboratory workforce [7,29]. In addition, tasks requiring awkward arm/hand exertion positions, repetitive movements, or using computers were associated with pain, particularly in the lower back, which is aligned with previous reports [19,30]. Laboratory professionals adopt inappropriate postures at workstations while performing laboratory-related tasks that induce musculoskeletal pain, so increasing the awareness of the proper body position while performing each laboratory task is required to assist in limiting MSDs among the staff.

Furthermore, neck and upper back discomfort were highly reported among workers exposed to frequent moving of heavy loads (more than 15 kg) in the clinical laboratory settings. In the current study, a statistically significant association was observed between upper back pain and frequent moving of heavy loads, which was not statistically significant with MSDs in a study by M et al. [19]; however, a high prevalence of musculoskeletal pain was documented among laboratory workers. In addition, this study found an association between shoulder and lower back pain and pipetting among technicians, consistent with a study that identified similar pain associated with pipette work in laboratory practices [15,21,30]. This could be due to standing or sitting pipetting in awkward postures, including forward bending of the neck, shoulder abduction, and elbow flexion without back support for a long period that put excessive strain on the back and shoulders.

This study encountered several limitations, including the nature of the cross-sectional study design, which limits the ability to determine causal associations among variables. Also, the study was limited to one city in Saudi Arabia, which may limit the generalizability of the study results to other cities, so future research is suggested to capture a snapshot of a wider geographical area in Saudi Arabia. Additionally, this study utilized the MSDs in any body region for the last 12 months to provide an initial explanation and broader insight into the overall MSD burden among laboratory technicians. However, future studies should incorporate time-specific analyses with body regions to provide a more comprehensive explanation of MSD patterns among technicians. The study reported the participants’ responses using a self-reported survey that may result in response bias. For future research, objective measuring assessments of the occupational environment may help to assess the ergonomic risk factors in medical laboratory settings. Lastly, the sample size and distribution did not allow the application of multivariate logistic regression without risking unstable estimates. Consequently, future studies should incorporate multivariate regression with larger and more diverse samples to better isolate independent risk factors for MSDs and account for potential confounders.

## 5. Conclusions

Work-related risk factors such as time spent standing or sitting, working in awkward positions, repetitive movements, or using computers were significantly associated with the prevalence of MSD. The findings indicated the significance of ergonomic adjustments, educational instruction, and proficient demonstration of corrective posture during routine laboratory tasks, ultimately seeking to diminish the prevalence of these disorders. Also, it is recommended to enforce the occupational health and safety policy in laboratory settings to foster secure working environments. Periodic ergonomic assessment and support from the occupational health and safety staff could reduce MSDs and improve technician-specific body ergonomics.

## Figures and Tables

**Table 1:** The distribution of sociodemographic and occupational variables (n = 167).

Variable		Frequency N	Percentage %
Gender	Male	80	47.9
	Female	87	52.1
Nationality	Saudi	143	85.6
	Non-Saudi	24	14.4
Age (in years)	20–30	72	43.1
	31–40	79	47.3
	41–50	7	4.2
	51–60	9	5.4
Experience (in years)	Less than 1 year	51	30.5
	1–5 years	28	16.8
	5–10 years	34	20.4
	More than 10 years	54	32.3
Body Mass Index (BMI)	Underweight	7	4.2
	Healthy	79	47.3
	Overweight	54	32.3
	Obese	27	16.2

**Table 2:** Musculoskeletal disorders prevalence during the past 7 days and 12 months (n = 167).

	12 Months Prevalence of MSDs (Percentage %)	7 Days Prevalence of MSDs (Percentage %)
Neck	48.5	37.3
Shoulder	40.7	25.7
Elbow	13.2	7.20
Wrist/hand	28.1	12.6
Upper back	33.5	21.0
Lower back	52.1	33.5
Hips/thighs	16.8	9.00
Knees	32.3	18.6
Ankles/feet	31.1	19.8
Any body part	71.3	58.1

**Table 3:** The results of the chi-square test for sociodemographic and occupational variables with MSDs in the last 12 months (n = 167).

		Presence of MSDs in the Past Year		
Variable		No (48)	Yes (119)	*p*-Value
Gender	Male (80)	30.0%	70.0%	0.431
	Female (87)	27.6%	72.4%	
Nationality	Saudi (143)	25.9%	74.1%	0.043 *
	Non-Saudi (24)	45.8%	54.2%	
Age	20–30 (72)	27.8%	72.2%	0.338
	31–40 (79)	26.6%	73.4%	
	41–50 (7)	28.6%	71.4%	
	51–60 (9)	55.6%	44.4%	
Experience	Less than 1 year (51)	31.4%	68.6%	0.024 *
	1–5 years (28)	21.4%	78.6%	
	5–10 years (34)	11.8%	88.2%	
	More than 10 years (54)	40.7%	59.3%	
BMI	Underweight (7)	28.6%	71.4%	0.212
	Healthy (79)	35.4%	64.6%	
	Overweight (54)	25.9%	74.1%	
	Obese (27)	14.8%	85.2%	

* Significant at the 0.05 level.

**Table 4:** Pearson chi-squared tests for MSDs in the past 12 months according to job nature.

	Presence of MSDs at Any Site				
		No (48)	Yes (119)		
Job Nature		No.	No.	Chi-Square	*p*-Value
Standing for long periods	No	24	32	8.195	0.004 **
	Yes	24	87		
Sitting for long periods	No	34	65	3.724	0.038 *
	Yes	14	54		
Working in the same position for long periods	No	32	44	12.160	0.000 **
	Yes	16	75		
Tasks requiring awkward position	No	38	76	3.696	0.039 *
	Yes	10	43		
Tasks requiring arm/hand exertion	No	34	53	9.477	0.002 **
	Yes	14	66		
Tasks requiring repetitive movements	No	32	51	7.756	0.004 **
	Yes	16	68		
Work using computers	No	17	34	0.756	0.246
	Yes	31	85		
Using comfortable chairs and disks	No	30	77	0.072	0.461
	Yes	18	42		
Frequently moving heavy loads (more than 15 kg)	No	43	96	1.946	0.120
	Yes	5	23		
Using microscope	No	28	64	0.286	0.359
	Yes	20	55		
Using microtome	No	40	104	0.475	0.322
	Yes	8	15		
Using biosafety cabinet	No	31	82	0.292	0.357
	Yes	17	37		
Pipetting for long periods	No	32	68	1.291	0.168
	Yes	16	51		

* Significant at the 0.05 level; ** Significant at the 0.01 level.

## Data Availability

Dataset available on request from the authors.
